# Mapping the structure of perceptions in helping networks of Alaska Natives

**DOI:** 10.1371/journal.pone.0204343

**Published:** 2018-11-12

**Authors:** Hsuan-Wei Lee, Miranda Melson, Jerreed Ivanich, Patrick Habecker, G. Robin Gauthier, Lisa Wexler, Bilal Khan, Kirk Dombrowski

**Affiliations:** 1 Department of Sociology, University of Nebraska, Lincoln, NE, United States of America; 2 Community Health Education, University of Massachusetts, Amherst, MA, United States of America; Queen Mary University of London, UNITED KINGDOM

## Abstract

This paper introduces a new method for acquiring and interpreting data on cognitive (or perceptual) networks. The proposed method involves the collection of multiple reports on randomly chosen pairs of individuals, and statistical means for aggregating these reports into data of conventional sociometric form. We refer to the method as “perceptual tomography” to emphasize that it aggregates multiple 3rd-party data on the *perceived* presence or absence of individual properties and pairwise relationships. Key features of the method include its low respondent burden, flexible interpretation, as well as its ability to find “robust intransitive” ties in the form of perceived *non*-edges. This latter feature, in turn, allows for the application of conventional balance clustering routines to perceptual tomography data. In what follows, we will describe both the method and an example of the implementation of the method from a recent community study among Alaska Natives. Interview data from 170 community residents is used to ascribe 4446 perceived relationships (2146 perceived edges, 2300 perceived non-edges) among 393 community members, and to assert the perceived presence (or absence) of 16 community-oriented helping behaviors to each individual in the community. Using balance theory-based partitioning of the perceptual network, we show that people in the community perceive distinct helping roles as structural associations among community members. The fact that role classes can be detected in network renderings of “tomographic” perceptual information lends support to the suggestion that this method is capable of producing meaningful new kinds of data about perceptual networks.

## Introduction

The way that people are classified into relational groups by knowledgeable outsiders has its own reality, a reality that says as much about where these outsiders perceive social fault lines to lie as it does the presence or absence of particular relationships [1]. In this article we present a strategy to recover some of the “heuristics” [2] people implicitly use to classify the relationships of others in their community via perceptual tomography—multiple reports on the presence or absence of social ties between randomly selected pairs of actors in a community. Following Krackhardt, such approaches are commonly referred to as cognitive social structures [3].

Although researchers have consistently returned to the concept of cognitive social structures [4–6], this type of work has often remained limited to questions of ego network recall, role perception and informant reliability [7], while most social network field studies continue to rely on “name generator” style elicitation techniques [8]. In the method adopted here, we combine network sampling approaches with third party reporting: randomly chosen individuals are shown a random sample of photographs from a group to which they belong and asked to bin the photographs according to whether or not the individuals in the photographs are “close to one another”. Following this, reports on perceptions of the social characteristics of these same individuals are gathered from the same respondent. Together, these data can be used to infer perceived ties (or the absence of ties) and the perceived attributes of those involved. Such a method offers a number of advantages, not least of which is that it simplifies what are otherwise complex issues of network sampling [9].

When social scientists rely on self-reported ties, sampling must take into account the network characteristics of those involved [10]. However, network structure is not normally known ahead of time—indeed, discovery of such information is usually the point of undertaking the survey. Under the scenario described here, it is assumed that any member of the community can report on the presence or absence of a tie between a randomly selected pair of other community members who are previously known to them—with some degree of error—in ways that reveal socially significant perceived relationship types and similarities among those thought to fill those categories [1]. When our interest is in meaningful perceptions of social structure, attention to the results of relationship perceptions allows us to sample widely from the population and collect multiple reports on any given random pair from a variety of “network angles”. We refer to this approach as perceptual tomography, as *multiple* 3rd party reports from a range of social positions within a network are aggregated to provide a picture of the perceived social topology.

In addition to the sampling advantages, two other possible benefits arise from the use of perceptual tomography. The first is the opportunity to collect a large amount of network data relatively easily. In ego-network elicitation methods, a respondent is limited to reporting on a fixed number of ties (i.e. his/her individual degree). When reporting on ties between other community members, it is possible to report on a high number of pairs in a short period of time though “binning”. In the example described below, individuals could easily bin 40 randomly chosen individuals and report on their characteristics in 10 minutes or less. Such an exercise produces reports on up to 40 × 40 ties. This is far greater than most ego network interviews could be expected to produce, and in a much shorter time. Additionally, third party reporting allows for the collection of information on their perceptions of the presence or absence of social ties, that individuals may not want to reveal about themselves. Individual reliability on tie reporting has been discussed at various points in the history of social network analysis [11], almost always pointing to the conclusion that such processes introduce hidden forms of reporting error into SNA data [12]. By relying on multiple reports from ostensibly un-invested third parties, we move away from a reliance on potentially highly subjective data sources.

Such an approach raises a number of challenges, however. Multiple reports on perceptions of the presence or absence of ties between a given pair necessarily raises the possibility of error in reporting and conflicting opinions about the tie. Similar issues are raised when we seek to determine perceptions of individual attributes via similar third party reporting. In both situations, inferring perceived pairwise relationships (or lack thereof) and perceived individual attributes (or lack thereof) with a rigorous sense of confidence, requires very different approaches than those that rely on self-reports. This is especially true where the number of reports may vary considerably across both pairs and attributes. These methods below yield data in conventional sociometric format, while accounting for differing numbers and discordant reports across pairs and individuals (a result of the random selection of photographs shown to a respondent). They also offer means for raising or lowering the confidence threshold used to determine the presence or absence of a perceived tie, or a perceived individual attribute. This allows for greater flexibility in situations where more general yet rigorous formalizations of perceptual networks are required.

A final benefit to the methods introduced here—and a central feature of the analysis that follows—is that they allow researchers to infer the presence of “robust intransitive” ties: these are pairs where there is a strong statistical reason to believe that a perceived tie is unlikely, given the number of reports received on a the pair (relative to a mathematically justifiable threshold value). As demonstrated below, the definitive absence of a perceived tie allows for balance clustering approaches in places where block modeling or other common equivalence approaches are less definitive.

## Background

### Network sampling, edge elicitation, and respondent reliability

The range of social network data collection methods is vast and continues to grow. From early anthropological studies of social interactions [13, 14] to contemporary sociological questionnaires and structured interviews [15], to the mining of existing relational data from already existing data sources [16, 17], the means for assembling relational data vary widely. Unless a population is known and bounded (e.g., classrooms of students), it is often difficult to obtain specific information on personal ties from all of the individuals in a naturally occurring population [18]. As noted by Frank [19], under such conditions, network sampling could provide an important alternative provided means for analyzing sampled data are available that can account for sampling-based uncertainty [20, 21]. Ego network research provides a number of sampling options [22], and examples of large scale nationally-representative ego network data collection include the General Social Survey (GSS) [23, 24] and the National Longitudinal Study of Adolescent to Adult (Add Health) [25, 26]. Guidelines for analysts working with whole networks have been proposed as well [27, 28], but considerable uncertainty remains. Respondent driven sampling [29, 30], snowball sampling [31], convenience sampling [32], and web-based sampling [33] have been employed in network studies (see [9] for a recent review), and a range of simulation experiments have been performed to discover the effects that missing or sampled data may have had on the overall topological characteristics of the graphs involved [34–36]. Considerable work remains to be done, however, as the unique dependency structure of tie data often makes “missing at random” assumptions problematic [35, 37].

Leaving aside sampling, incomplete data can result from other sources as well, including respondent error or fatigue. Ego network elicitation that asks respondents to describe the personal attributes and local social connections for a long list of alters can prove highly burdensome [23], and surveys looking to obtain alter-alter relations and alter specific characteristics (i.e., name interpreter questions) add to that burden [38]. Further, one of the dominating concerns of social network scientists is the reliability of respondent social networks given different conceptual processes [39] and demographic differences [40]. Simple differences in how alters are thought of and described can lead to high variation in reports—concerns magnified when researchers rely entirely on the endpoints of a tie to justify its presence [41].

Unlike traditional ego centric (two dimensional) network data collection, cognitive social structures are three dimensional network structures. As described by [3], the reports on the nature of alter-alter social relationships were thought interesting both for what they told us about the social structure, and for what they told us about the social-conceptual processes of the reporting party. This approach to network data structure has proven to be useful in several meaningful ways. First, cognitive network-style elicitation can be used when large scale data collection is not possible/feasible [3]. Second, these data provide a different theoretical perspective to understand relationships—the core of social network analysis [42]. Lastly, [4] has shown the utility of using cognitive social networks as a fruitful tool for exploring multilevel organization dynamics.

While potentially quite novel, gathering large samples of cognitive social structures can be difficult due the fact that data collection is cognitively expensive for respondents. This limitation to the cognitive social structure approach has been an enduring problem for researchers who desire to use this approach but work with samples that are large [3].

### Community detection and balance clustering

Many complex networks have natural community structures that are crucial to understanding their network properties. Here the basic objective is to classify objects into different groups such that nodes within the same groups are similar. Classical block modeling approaches draw on matrix permutation and density measures [42], while many recent global graph partition approaches borrowing ideas from statistical physics [43–45]. The latter make use of a concept modularity, or a measurement of the strength of division of a network into modules. Intuitively, networks with high modularity have dense connections between the entities within communities but sparse connections between entities in different communities. These techniques have the benefit of being usable across weighted [46], signed [47], and multilayer networks [48].

Balance clustering of ties leverages the presence of both positive, negative, and null ties. This work draws on structural balancing theories of cognitive fields introduced by [49]. Heider examined triads, between a Person [P], an Other Person [O], and an Object or Topic [X]. Signed ties were introduced, which are traditionally defined as either positive (e.g. liking, loving, supporting) or negative (e.g. disliking, hating, opposing) edges. Heider posited a balanced triad state which “exists if all parts of a unit have the same dynamic character (i.e., if all are positive, or all are negative), and if entities with different dynamic character are segregated from each other” [49], and hypothesized that people prefer balanced triadic relationships in order to avoid stress or tension. Cartwright and Harary [50] applied this notion to triads of persons, allowing its wider application in sociometric contexts. Here the process of actors forming and/or dropping signed ties could be seen as a consistent micro-social processes that would result in larger, observable social structures. Under such conditions, the nodes of a completely balanced network could be partitioned into two classes in which all of the positive ties exist within the classes and all of the negative ties are between them. Davis [51] later expanded balancing to include multiple classes, providing a flexible framework that, according to Dorian and Mrvar “linked the micro-processes of tie formation and change within triads to a statement about the overall group structure for balanced networks” [52]. As Dorien et al. note, one result of this theory is an explanation for the commonplace observation that a friend of a friend will be a friend; a friend of an enemy will be an enemy; an enemy of a friend will be an enemy; and an enemy of an enemy will be a friend [53].

Balance clustering remains rare in contemporary social network analysis, in large part because most social network data lacks negative tie designations necessary to apply the original theorem. Recently, however, balance theories have been applied via machine-learning models of online datasets [54]. Drawing on Cartwright and Harary [50], this technique makes use of assumed triad balance to predict the presence and absence of previously unknown ties with high accuracy. As Leskovec et al. note: “In the same way that link prediction is used to infer latent relationships that are present but not recorded by explicit links, the sign prediction problem can be used to estimate the sentiment of individuals toward each other, given information about other sentiments in the network” [54]. These authors show that a sample of negative and positive edges can be used to posit the presence of undiscovered ties—both positive and negatives. From these results they conclude that there was, “a significant improvement to be gained by using information about negative edges, even to predict the presence or absence of positive edges” [54]. Echoing Cartwright and Harary, they conclude that “it is often important to view positive and negative links…as inter-related, rather than as distinct noninteracting features of the system.” “Others have questioned such blanket application of balance theories, including [52]. They cite blockmodels performed by Newcomb [55] as evidence that balance theory is often not sufficient to account for the presence of all negative ties in a network. Noting that negative ties seem to accumulate disproportionately around some parts of a network, Doreian concludes that “[t]he increased concentration of negative ties on some actors suggests differential dislike is either a more potent process than structural balance or is an unrecognized component of it” [52].

In what follows, we introduce a different approach to definition of negative ties and the utility of balance clustering in global graph partitioning. Here we use the notion of “robust intransitive ties”—pairs which are highly unlikely to be perceived as in-relationship, given the number and distribution of reports. In cases where we see strong statistical reason to believe a tie is highly unlikely, we signify this as a negative edge—distinct from positively inferred edges (also based on the number and distribution of reports) and null ties (for which there is a lack of statistical clarity one way or another). Importantly, robust intransitive ties are not standard negative ties in that they do not reflect reports of animosity of one node towards another. Rather, they are a diagnostic aid used in the partitioning of the network by allowing for more stringent, theory-based clustering criteria than is available from other group detection protocols.

### Helping relationships in Alaska Native communities

To demonstrate the feasibility of both a new means of cognitive network data elicitation and a method for ascribing sociometric data from the reports of a community sample, we discuss an example drawn from fieldwork among Alaska Natives in 2015. As part of a pilot project aimed at community readiness and social relationship building around substance abuse and suicide [NIH R34 MH096884], our team of two interviewers conducted 170 interviews in a northern Alaska Native community (for descriptive statistics, see S1 Table) of approximately 360 adults using a tablet-based survey employing Social Network Analysis through Perceptual Tomography (SNAPT) software. Data collection took place over seven days, with interviews averaging 10-20 minutes. Eligible participants were aged 12 or older and were current residents of the community. Recruitment into the project was enabled through peer referral sampling, wherein an initial batch of six interviews were conducted and each of those participants were given three coupons that were used to recruit other eligible participants. Interviews and recruitments were carried out recursively until a final sample of n = 170 was achieved. A participant received $20 for the initial interview and could earn and additional $5 for each of their referral coupons that resulted in a completed interview. All interviews were conducted in the break room of the local health clinic and were not scheduled ahead of time. Each participant was registered in a coupon-referral tracking software, completed a one-page demographic paper questionnaire, and then completed the SNAPT questionnaire on a tablet (see [56] for a full discussion of the project). For more information, please see the zip file study_data.zip associated with the manuscript.

The SNAPT questionnaire showed each participant 40 names or pictures of people drawn randomly from the list of all eligible participants in the town (compiled prior to the first interview from administrative sources and community volunteers). Participants were asked to sort that name/photos that appeared into one of three bins: (1) “Someone I am Close To,” (2) “Someone I Recognize/Know,” or (3) “Someone I Don’t Know.” Next, participants were shown all of the names/pictures from category (2) and asked to place the people they said they recognize/know (but were not close to) into clusters of people who were close to one another. For this task the participant could create up to five separate and mutually exclusive clusters. A name/picture could only be in one cluster. Next, for each of the clusters created, participants were asked to identify what sort of cluster this was from a list of different labels (family, friends, people who attend the same church, etc.). After labeling the cluster, participants had to describe the roles that people these clusters play in the community. Participants could choose multiple options from a list of 16 predesignated helping roles (i.e. helps young people, helps women who are having trouble at home, is a member of a respected family, etc. See Table 1: A1-16). Finally, the name/photos of individuals from bin (1) were shown and the participant was allowed to identify for each individual whether he/she played any of seven different personal helping roles. (These data do not pertain to group characteristics and are not relevant to this analysis).

**Table 1:** Attribute list.

Variable Name	Variable Description
A1	Makes positive changes in the community
A2	Helps young people in general
A3	Helps people with alcohol problems
A4	Helps women who are having trouble at home
A5	Helps men who are having trouble at home
A6	Helps elders who are having trouble at home
A7	Helps young people who are having trouble at home
A8	Helps people learn about traditional knowledge
A9	Gives money food or other needed things to people who need them
A10	Will correct a young person if he or she is doing something wrong
A11	Is a member of a respected family
A12	Act in ways that are good for the community
A13	Give good advice most of the time
A14	Are a positive influence on others in this community
A15	Are willing to help out people who are in need
A16	Helps people who tend to be left out

We summarize the basic descriptive statistics for the 170 sampled participants in the Supporting Information. Overall, the sample was mostly males (59%), with an average age of 35 years (with the youngest participant being 12 and the oldest participant being 89 years old). A sizable percentage of participants in our sample had a high school degree or less (87%). Additionally, over half (59%) said they make less than $400 a week. The average number of children and adults in their households were similar (2.42 and 2.92, respectively). We also computed a subsistence access scale as a local measure of socioeconomic status. To calculate this score, respondents were asked about their access to key hunting/subsistence tools including snow mobile/skidoo, cabins, and boats. Individuals could indicate they have no access (0), access but do not own (1), or that they owned (2) one or more resources in each of these areas. These three measures were summed for a total subsistence access score [57]. The average score was 2.50 out of a maximum score of 6.

## Inferring perceptual networks from the SNAPT process

The object of study is a population *P* having |*P*| = *n* individuals, each of which has been photographed in advance. Additionally (at the outset), as researchers, we have identified a set of *M* binary attributes of interest (e.g. “This person makes positive changes in the community”) that we seek to ascertain about the populations’ members. We assume that for each individual *p* ∈ *P* and attribute *a* in {1, …, *M*}, the individual either has attribute *a* or does not. We capture this by defining ground truth via a function *Q* where

$$Q\left(p,a\right)=\{\begin{array}{cc} +1 & \text{if}\;p\;\text{has}\;\text{property}\;a.\\ -1 & \text{if}\;p\;\text{does}\;\text{not}\;\text{have}\;\text{property}\;a. \end{array}$$ (1)

Our goal is to get a picture of the perceptual network structure of *P* and the perceived attributes of individuals therein (*Q*), using individuals’ perceptions of others as the penetrating wave by which tomographic images of network sections are obtained, and to subsequently synthesize these images into a quantitative description of the ensemble as a whole.

### Data collection

We sample a random subset *S* ⊆ *P* of size *m* ≤ *n*. Each sampled individual *s*_*i*_ ∈ *S* (where *i* = 1, … *m*) participates via a 3-step process. In **step 1**, subject *s*_*i*_ is shown a random subset of *k* photos *V*_*i*_ ⊆ *P*, |*V*_*i*_| = *k*, and asked to partition *V*_*i*_ into three disjoint bins: ($C_{1}^{i}$) those who are “close to me”, ($C_{2}^{i}$) those who are “not close to me but that I recognize” and ($C_{3}^{i}$) those that “I do not recognize”. The number of individuals that *s*_*i*_ recognizes (from *P*) is referred to as their “recognition degree” and denoted as $d\left(s_{i}\right)=\left|C_{2}^{i}\right|$. In **step 2**, subject *s*_*i*_ is asked to sort the photos in the second bin (“not close to me but that I recognize”) further by placing each of its $|C_{2}^{i}|$ members into one of *B* clusters, according to the instruction “Place people who you think are close to each other into the same cluster”. In **step 3**, subject *s*_*i*_ is asked to give their “opinion” on whether each member $s_{j}\in C_{2}^{i}$ has attribute *a* (i.e. *P*(*s*_*j*_, *a*) = +1), or does not (i.e. *P*(*s*_*j*_, *a*) = −1), for each *a* in {1, …, *M*}.

For each *s*_*j*_ ∈ *S*, we denote the subjects who were shown and recognized *s*_*j*_ as

$$O_{j}=\left\{s_{i}\in S\;|\;s_{j}\in C_{2}^{i}\right\},$$ (2)

and then for each *u* ∈ *O*_*j*_ and attribute *a* in {1, …, *M*}, we define

$$o\left(u,a,s_{j}\right)=\{\begin{array}{cc} 1 & \text{if}\;u\;\text{reported}\;\text{the}\;\text{opinion}\;\text{that}\;s_{j}\;\text{has}\;\text{property}\;a.\\ 0 & \text{if}\;u\;\text{reported}\;\text{the}\;\text{opinion}\;\text{that}\;s_{j}\;\text{does}\;\text{not}\;\text{have}\;\text{property}\;a. \end{array}$$ (3)

### Ascribing network ties

The sociometric analysis of the data focuses on the clustering of known (but not “close to”) community residents from bin $C_{2}^{i}$ where *i* ranges in {1, …, *m*}. In interpreting the data collected (see above), the basic challenge is (i) quantifying whether (or when) co-placement of a pair of individuals into the same cluster may be taken as a significant evidence of a perceived social relationship between the pair (or dismissed as failing to rise above random chance); and (ii) quantifying whether (or when) the placement of a pair of individuals into different clusters may be taken as a significant evidence of the *absence* of a perceived social relationship between the pair (or dismissed as failing to rise above random chance).

### Null model

Measuring significance in regards to (i) and (ii) above, requires that we specify a “null model” that quantifies the notion of random chance. In this work, we assume a null model N where each individual *s*_*i*_ ∈ *S* acts as follows:

1. In step 1, *s*_*i*_ recognizes other individuals *v* ∈ *P* (*v* ≠ *s*_*i*_) (we assume if individual *s*_*i*_ sees their own photo, they don’t put the photo into the $C_{2}^{i}$ group) with a fixed uniform probability *γ* ∈ [0, 1], and thus recognition degree $d\left(s_{i}\right)=\left|C_{2}^{i}\right|$ follows a Bernoulli distribution with bias *γ*. The specific value of *γ* in the null model will be described in a later section.
2. In step 2, *s*_*i*_ acts *blindly*, sorting $C_{2}^{i}$ by placing each photo therein into a random cluster, chosen independently and uniformly at random from the *B* options.
3. In step 3, *s*_*i*_ opines randomly about whether or not individual $s_{j}\in C_{2}^{i}$ has property *a* (for *a* = 1 …, *M*). More precisely, *s*_*i*_ expresses the opinion that *Q*(*s*_*j*_, *a*) = +1 (i.e. that *o*(*u*, *a*, *s*_*j*_) = +1) with a fixed uniform probability *β*_*a*_ ∈ [0, 1]—and expresses the opinion that *Q*(*s*_*j*_, *a*) = −1 otherwise. The specific value of *β*_*a*_ in the null model is described in a later section.

We want to understand the distribution of outcomes when the null model N is engaged. Towards this, we introduce random variables *X*(*v*_0_, *v*_1_) and *Y*(*v*_0_, *v*_1_) for each pair of distinct *v*_0_, *v*_1_ ∈ *P*. Here *X*(*v*_0_, *v*_1_) (resp. *Y*(*v*_0_, *v*_1_)) is defined to be the number of individuals that recognized both *v*_0_ and *v*_1_ *and* placed them in the *same* (resp. *different*) clusters. Since all subjects behave uniformly in the null model, these $2\left(\begin{array}{c} m\\ 2 \end{array}\right)$ random variables enjoy identical distributions; in what follows we will, for this reason, frequently refer to them indistinguishably as simply *X* (resp. *Y*).

Each individual *s*_*i*_ ∈ *S* recognizes precisely *r* (0 ≤ *r* ≤ *n* − 1) individuals from *P* with probability

$$z_{r}=\left(\begin{array}{c} n-1\\ r \end{array}\right)\gamma^{r}{\left(1-\gamma \right)}^{n-r-1}.$$ (4)

For integer *r* ∈ [0, *n* − 1], if $|C_{2}^{i}|=\ell ⩾2$, then for integer *ℓ* ∈ [2 ∨ (*k* + *r* − *n*), *r* ∧ *k*]:

$$Prob\left[\left|C_{2}^{i}\right|=\ell \right]=\frac{\left(\begin{array}{c} r\\ \ell \end{array}\right)\left(\begin{array}{c} n-r\\ k-\ell \end{array}\right)}{\left(\begin{array}{c} n\\ k \end{array}\right)}.$$ (5)

The probability that both $v_{0},v_{1}\in C_{2}^{i}$ is

$$\left(\begin{array}{c} \ell \\ 2 \end{array}\right)/\left(\begin{array}{c} n\\ 2 \end{array}\right).$$ (6)

Given $|C_{2}^{i}|=\ell$, and assuming that both $v_{0},v_{1}\in C_{2}^{i}$, the probability that respondent *s*_*i*_ (acting according to the null model) would place both *v*_0_, *v*_1_ in the same cluster is 1/*B*. Thus, a fixed *s*_*i*_ ∈ *S* will recognize both *v*_0_, *v*_1_ and place them in the same cluster with probability

$$p=\left(\frac{1}{B}\right)\cdot \sum_{r=0}^{n-1}\sum_{\ell =2\vee k+r-n}^{r\wedge k}\left(\begin{array}{c} n-1\\ r \end{array}\right)\gamma^{r}{\left(1-\gamma \right)}^{n-r-1}\frac{\left(\begin{array}{c} r\\ \ell \end{array}\right)\left(\begin{array}{c} n-r\\ k-\ell \end{array}\right)}{\left(\begin{array}{c} n\\ k \end{array}\right)}\frac{\left(\begin{array}{c} \ell \\ 2 \end{array}\right)}{\left(\begin{array}{c} n\\ 2 \end{array}\right)}.$$ (7)

and will recognize both *v*_0_, *v*_1_ and place them in different clusters with probability

$$q=\frac{B-1}{B}\sum_{r=0}^{n-1}\sum_{\ell =2\vee k+r-n}^{r\wedge k}\left(\begin{array}{c} n-1\\ r \end{array}\right)\gamma^{r}{\left(1-\gamma \right)}^{n-r-1}\frac{\left(\begin{array}{c} r\\ \ell \end{array}\right)\left(\begin{array}{c} n-r\\ k-\ell \end{array}\right)}{\left(\begin{array}{c} n\\ k \end{array}\right)}\frac{\left(\begin{array}{c} \ell \\ 2 \end{array}\right)}{\left(\begin{array}{c} n\\ 2 \end{array}\right)}.$$ (8)

The analysis above is with respect to data from a fixed *s*_*i*_ ∈ *S*. In considering data collected from the sample set *S* in aggregate, we observe that in the null model, a binomial distribution governs the probability that exactly *w* individuals place both *v*_0_, *v*_1_ into $C_{2}^{i}$ and then put them into the *same* cluster:

$$Prob\left[X=w\right]=\left(\begin{array}{c} m\\ w \end{array}\right)\cdot p^{w}{\left(1-p\right)}^{m-w}.$$ (9)

and analogously, the probability that exactly *w* individuals place both *v*_0_, *v*_1_ into $C_{2}^{i}$ and then place them in *different* clusters, is given by:

$$Prob\left[Y=w\right]=\left(\begin{array}{c} m\\ w \end{array}\right)q^{w}{\left(1-q\right)}^{m-w}.$$ (10)

### Parameterizing the null model

In what follows, the distributions of outcomes (i.e. *X* and *Y*) when the null model N is engaged, will be used to define criteria by which to decide the significance of outcomes observed in a non-null model D—e.g. one that is based on concrete empirical data such as the Northern Alaskan community network. For *X* and *Y* to be fully defined and computable, however, *M* + 5 free parameters of the null model need to be specified: *n*, *m*, *k*, *B*, *γ*, and *β*_*a*_ where *a* ranges in {1, …, *M*}. In the Northern Alaskan community network, we have *n* = 393 and *m* = 172. The current version of the SNAPT software implements *B* = 5, and *k* = 40.

The remaining *M* + 1 free parameters (*γ*, and *β*_*a*_ where *a* ranges in {1, …, *M*}) are tuned so that key first order statistics of the outcomes exhibited by the null model N agree with the corresponding sample statistics of the outcomes observed from D. First, to ensure that expected number of positive assertions in N concerning property *a* will agree with the actual number observed in D we set

$$\beta_{a}=\frac{\sum_{s_{i}\in S}\sum_{u\in O_{i}}o\left(u,a,s_{i}\right)}{\sum_{s_{i}\in S}\left|O_{i}\right|}$$ (11)

for each *a* in {1, …, *M*}. Additionally, to ensure that expected number of *γ* in N will agree with the actual number observed in D we set

$$\gamma =\frac{\sum_{s_{i}\in S}d\left(s_{i}\right)}{mk}=\frac{\sum_{s_{i}\in S}\left|C_{2}^{i}\right|}{mk}.$$ (12)

For Northern Alaskan community network, *γ* ≈ 0.7622. Note that in Eqs (11) and (12), the left hand-side is a free parameter of N, while the right-hand side is an expression evaluated in D. This completes the specification of the null model.

With a fully specified null model N in hand, we are able to make concrete numerical computations concerning the distributions of *X* and *Y*. We find that with 98.3% confidence, two random subjects will be recognized and placed in the same cluster by 1 or fewer subjects, and similarly, with 95.4% confidence, two random subjects will be recognized and placed in different clusters by 2 or fewer subjects. To establish integer cutoffs, we define Δ^+^(0.95) to be the minimum integer *w* for which *Prob*[*X* < *w*] ≥ 0.95, and Δ^−^(0.95) to be the minimum *w* for which *Prob*[*Y* < *w*] ≥ 0.95. In the null model (parameterized by *n*, *m*, *B*, *k*, *γ* as above) we find:

$$\Delta^{+}\left(0.95\right)=2,$$ (13)

$$\Delta^{-}\left(0.95\right)=3.$$ (14)

**Fig 1:**
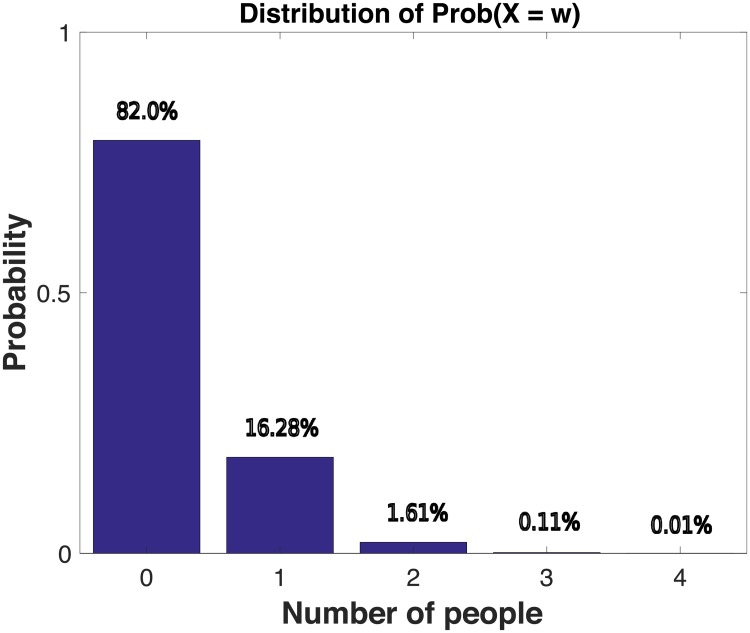
Sample distribution of *X* in a Northern Alaskan community. Here on the x-axis is the number of subjects that put a certain pair (*v*_0_, *v*_1_) among all pairs of distinct subjects in the same cluster and y-axis is the corresponding probability under the assumption of the null model N.

**Fig 2:**
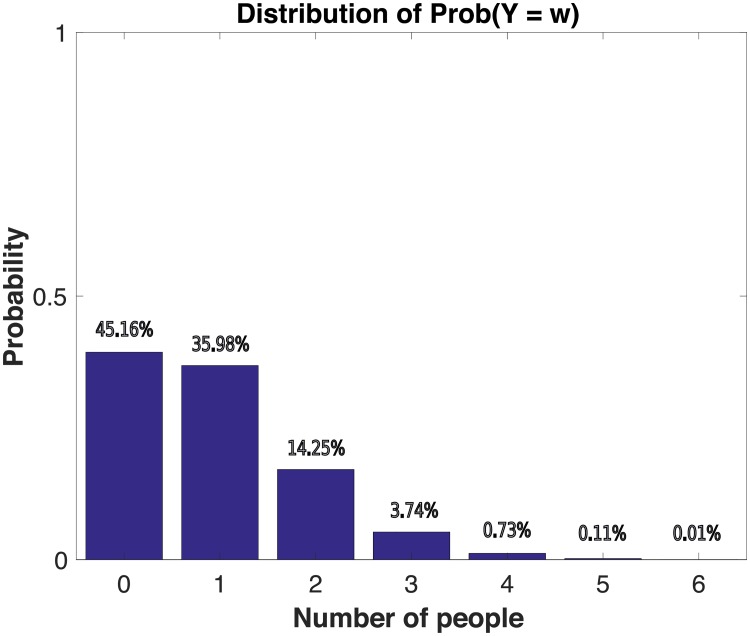
Sample distribution of *Y* in a Northern Alaskan community. Here on the x-axis is the number of subjects that put a certain pair (*v*_0_, *v*_1_) among all pairs of distinct subjects in the same cluster and y-axis is the corresponding probability under the assumption of the null model N.

Thus, in the Northern Alaskan community network, whenever we observe that ≥ 2 or more subjects put a pair photos in the same cluster, we consider it to be significant evidence that this pair was perceived to be in social relationship. On the other hand, when we observe that ≥ 3 subjects put a pair photos into different clusters, we consider this to be significant evidence that the pair was perceived to *not* be in social relationship. Fig 1 (resp. Fig 2) shows the empirical histogram of *X*(*v*_0_, *v*_1_) (resp. *Y*(*v*_0_, *v*_1_)) over all distinct pairs *v*_0_, *v*_1_ of subjects sampled from the Northern Alaskan community. We see that 1.73% of the pairs placed in the same cluster were statistically interpretable as being relationship, while 4.59% of the pairs placed in the different clusters were statistically interpretable *not* being in relationship (at the 95% confidence level).

### Ascribing perceived attributes

Similar challenges arise when we wish to interpret multiple and potentially conflicting reports regarding perceived attributes of individuals in the community. The basic challenge here is (i) quantifying whether (or when) the 3rd-party attribution of properties (resp. lack thereof) to individuals should be taken as a significant evidence of the individual being perceived to have (or lack) an attribute, and when such 3rd-party attributions should be dismissed as failing to rise above what might be expected by sheer chance.

Towards this, for each *s*_*i*_ ∈ *S* and *a* ∈ {1, …, *M*} we introduce a random variable *Z*(*s*_*i*_, *a*) whose value is the number of individuals that gave an affirmative opinion when questioned about whether *s*_*i*_ is perceived to have attribute *a*. In the null model, *Z*(*s*_*i*_, *a*) follows a binomial distribution

$$Z\left(s_{i},a\right)∼Bin(\beta_{a},|O_{i}\left|\right).$$ (15)

Note that the expected number of positive opinions on the question is

$$E\left[Z\left(s_{i},a\right)\right]=\beta_{a}\left|O_{i}\right|.$$ (16)

In the (non-null) model D, for each each individual *s*_*i*_ and attribute *a*, we seek to determine estimate $\tilde{Q}\left(s,a\right)$, thereby deciding whether *s*_*i*_ is perceived to have attribute *a* (or not). Towards this, we first determine the number of 3rd-party opinions (in D) supporting the assertion that *s*_*i*_ has property *a*:

$$f\left(s_{i},a\right)=\sum_{u\in O_{i}}o\left(u,a,s_{i}\right)$$ (17)

and then compare *f*(*s*_*i*_, *a*) to *β*_*a*_|*O*_*i*_|, the number of positive votes we would expect to find in the null model. If *f*(*s*_*i*_, *a*) is greater than *β*_*a*_|*O*_*i*_|, we do a right-tailed hypothesis test to determine whether the difference is significant at the *α* = 95% confidence level; if it is, we estimate $\tilde{Q}\left(s_{i},a\right)=+1$ “(have)”. Conversely, If *f*(*s*_*i*_, *a*) is less than *β*_*a*_|*O*_*i*_|, we do a left-tailed hypothesis test to determine whether the difference is significant at the *α* = 95% confidence level; if it is, we estimate that $\tilde{Q}\left(s_{i},a\right)=-1$ “(not have)”. It is also possible that some individual/attribute pairs may pass both significance tests or fail to meet either significance test; for these we arrive at a neutral estimate $\tilde{Q}\left(s_{i},a\right)=0$ “(inconclusive)”.

In the Northern Alaskan community network, we had *N* = 393 individuals who were observed by the opinion-givers and we had *M* = 16 attributes (see Table 1). Each community attribute was coded one of three values. An individual could be assigned a −1 or +1 depending on the number of affirmations or the lack of affirmations assigned to that individual by respondents during the data collection. Those not meeting either threshold were assigned a value of “0”. The number respondents found to be perceived to “have”, “not have”, or be “inconclusive”, with respect to each attribute can be found in Table 2.

**Table 2:** Summary of the number of individuals found to be perceived to “have”, “not have” or be “inconclusive” for a particular attribute at a significance level of 95% in the Northern Alaskan community network.

Attribute	(have)	(not have)	(inconclusive)
A1	47	6	340
A2	39	5	349
A3	12	15	366
A4	11	18	364
A5	11	15	367
A6	46	2	345
A7	29	6	358
A8	46	5	342
A9	56	8	329
A10	90	3	300
A11	120	1	272
A12	111	3	279
A13	55	5	333
A14	71	4	318
A15	109	1	283
A16	152	0	241

## Case study in balance clustering of perceptual networks with perceived attributes

The method described above produces a perceptual network of perceived ties (and non-ties) whose nodes are perceived to have (or lack) certain attributes. The question of whether such a process is capable of producing meaningful data remains open. As a step toward establishing the usefulness of the SNAPT method, we describe a global partitioning of thin network (produced in section 3) via balance clustering. The resulting structural classes are then analyzed to determine whether and to what extent they contain disproportionate numbers of individuals that play specific helping roles in the community. A key feature of the group detection protocol is the ability of the SNAPT method to produce data on “robust intransitives”: pairs of individuals between whom a perceived network tie was seen to be highly unlikely.

### Balance classes

SNAPT data collection and the above analytical protocol produced a signed network of 393 nodes with 4446 perceived edges. Of these edges, 2146 of them are perceived as positive and 2300 of them are perceived as negative. The average degree of a node (including both positive and negative edges) is approximately 23. Summary statistics of the network consisting of just positive edges and the network consisting of just negative edges are given in Table 3.

**Table 3:** Summary statistics of the network with positive edges (positive network) and the network with negative edges (negative network).

	Positive network	Negative network
Number of nodes	386	380
Number of edges	2146	2300
Mean degree	5.56	6.05
Diameter	6	6
Mean distance	2.79	2.70
Transitivity	0.11	0.09
Mean closeness	0.00021	0.00015
Mean eigenvector centrality	0.21	0.21
Mean betweenness	353.66	333.61

We chose a balancing method proposed by [58], which draws from idealized blockmodels informed by structural balance. The result is a partitioning of the graph with a blockmodel structure(s) of signed networks closest to the ideal form implied by structural theorems [50, 59]. We utilized the *Pajek 4.10* [60] program to implement the balance partitioning. The Pajek balance algorithm outputs the number of solutions/partitions that achieve the minimum number of inconsistencies/errors *R*, referred to here as the “balance score”. *R* is calculated with the formula of *R* = *αN*_*c*_ + (1 − *α*)*P*_*c*_, where *P*_*c*_ is a count of the positive signed edges found between nodes in different clusters, and *N*_*c*_ is a count of the negative signed edges between nodes in the same cluster. The *α* term allows for the differential weighting of unbalanced positive or negative ties. The lower the value of *R*, the more the partition obeys the balance prediction of [50]. The goal of the operation is to find a unique solution that minimizes the balance score.

The free parameters for the classification include the number of classes, the number of fitting optimization repetitions, the minimum number of nodes in a class, and the *α*-level used to determine the balance score. We varied the number of classes from three to nine using a minimum of three nodes per class and an *α*-level of 0.5. A minimum class size of three conforms to a minimal-sociologically meaningful group [61], while a value of *α* = 0.5 allowed robust intransitive ties to play a significant role in determining the final partition. Subsequent experiments with varying levels of *α* produced high numbers of solutions with only marginal improvements in the balance score. The optimization begins with a random partition containing the specified number of classes, and each repetition of the optimization begins with a new, randomly chosen partition as a basis for optimization. If the program finds several optimal solutions, all of them are reported [60].

**Fig 3:**
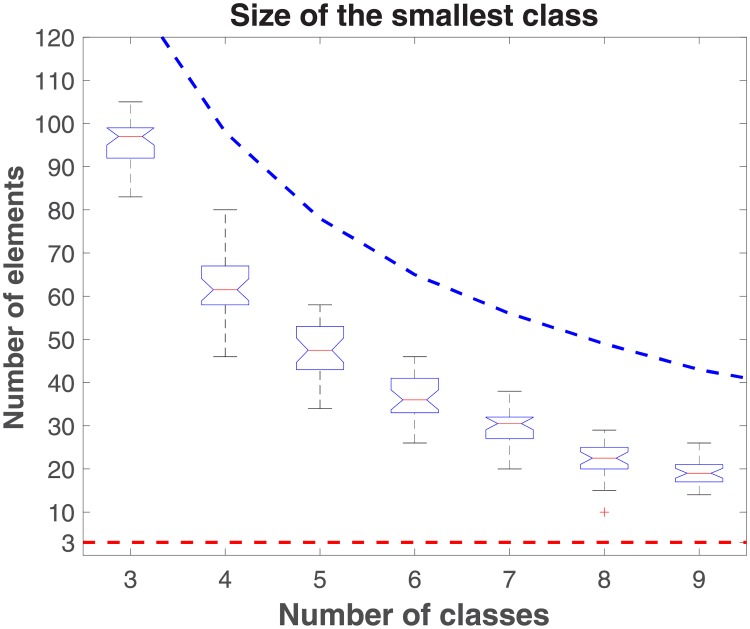
Boxplot of the size of the smallest class (given a designated number of classes) across 30 trials of 1000 repetitions, with *α* = 0.5. The theoretical upper bound for each experiment is the number of nodes that would appear in each class if all classes were the same size. The minimum class size was set at three. These results show that a minimum class size of three did not impinge on the optimization process.

**Fig 4:**
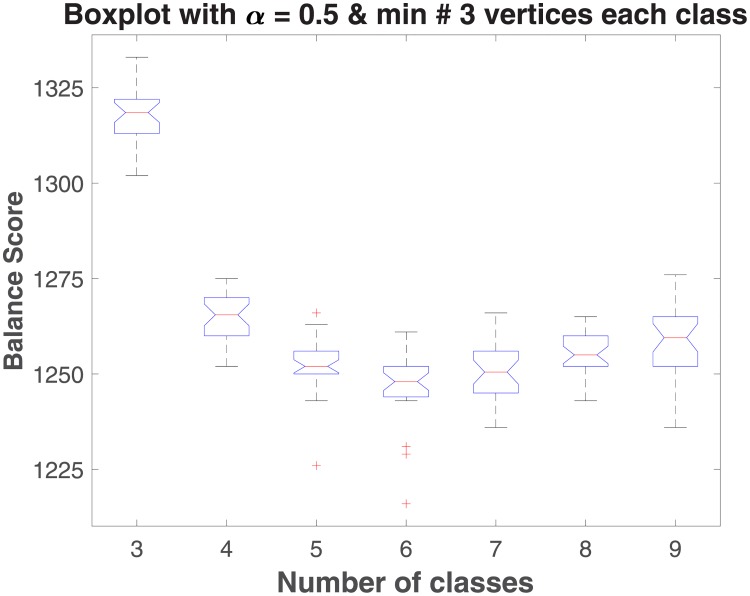
Boxplot of balance score versus number of classes as these are varied from three to nine. The concave shape indicates that the optimal number of classes, on the basis of balance score, is six.

To determine the optimal number of classes, we first varied the number of classes from three to nine and plotted the corresponding size of smallest class(es) (see Fig 3). These results showed that the minimum class size setting of three was not a significant determinant of class formation in our results. Next we sought to determine the optimum number of classes. Fig 4 shows the resulting balance scores as the number of classes is again varied from 3 to 9. The boxplot in Fig 4 manifests a concave up curve that reaches its minimum when the number of classes is equal to six. A second criteria for model selection was the desire for a unique solution (prompted in part by the fact that the Pajek algorithm is capable of discovering multiple, equivalent solutions based on balance score). Table 4 shows the results of variations in the number of classes from three to nine, the number of outliers (i.e. number of trials out of 30 that did not yield a unique solution), and the average number of solutions found in those outliers for a given number of classes. Here too, a model based on six classes was among the more optimal. As a final approach, then, we chose a six class model for the Northern Alaskan community network. The final result over 30 trials of 1,000 repetitions was a single unique solution of six classes, sized: 39, 42, 151, 71, 48, and 42 nodes, respectively.

**Table 4:** The results of experiments that varied the number of classes from three to nine, showing the number of outliers—the number of trials out of 30 that did not yield a unique solution. Row two shows the average number of solutions found in those outliers. The results indicate that a partitioning into either 4 or 6 classes is most likely to produce a unique solution.

Number of classes	3	4	5	6	7	8	9
Number of outliers	3	0	1	0	2	1	2
Averages of outliers	51	0	100	0	155.5	122	210

### Aligning the balance clustering results with attributes

Given the results of balanced clustering and the data on perceived attributes derived in the sections above, we now seek to assess possible relationships between balance cluster (class) membership and perceived attributes related to helping roles. Finding a relationship would imply that the structure of the network is influenced by homophily based on helping behaviors [62, 63]. Since each perceived attribute was coded as one of three mutually exclusive values -1 (“negative”), 0 (“inconclusive”), and 1 (“positive”), multinomial logistic regression analyses were appropriately employed [64] to estimate the probability of individuals in each cluster manifesting a perceived attribute (or lack thereof), comparing to a baseline reference.

Multinomial logistic regressions were carried for all 16 attributes. To illustrate, one of these regressions seeks to model the perceived attribute of “making positive changes in the community” in terms of class membership; it is presented in Table 5, while the complete results are shown in S2 Table. For the purposes of such analyses, classes were dummy coded, with membership in Class 3 being taken as attribute value 0. In Table 5 we see that membership in Class 2, (compared membership in Class 3), was significantly more likely to predict being perceived as making positive changes in the community. These results suggest that class membership derived from the SNAPT data collection and the edge/attribute synthesis process prove useful in discovering clusters of individuals who are perceived to play a particular helping role—documenting the existence of what Freeman once referred to as cognitive categories within structures of social affiliation [1]. The regression analyses of all 16 attributes are in S2–S17 Tables.

**Table 5:** Multinomial results illustration (A4: Makes positive changes in the community).

	*Dependent variable*:	
	Makes positive changes in the community^a^	
	(-1)	(1)
Class 1^b^	-8.542	-0.106
	(98.995)	(0.588)
Class 2^b^	-7.007	0.871 *
	(47.379)	(0.445)
Class 4^b^	0.048	-0.147
	(1.234)	(0.475)
Class 5^b^	0.511	-0.243
	(1.238)	(0.585)
Class 6^b^	1.313	-0.135
	(1.018)	(0.587)
Constant	-4.175 ***	-2.034 ***
	(0.713)	(0.258)
Akaike Inf. Crit.	355.913	355.913

*p<0.1;

**p<0.05;

***p<0.01

^a^—Reference category—“0”s

^b^—Reference category is Class 3

The full results show considerable predictive power for balance class membership and helping roles found in Table 1. Leaving aside those values or categories with low cell counts (see Table 2, significant relationships were found between class membership and those who make positive changes in the community (Class 2); help women who are having trouble at home (Class 5); help men who are having trouble at home (Class 1); help young people who are having trouble at home (Class 2); help people learn about traditional knowledge (Class 2); and give food or money to people who need it (Classes 2 & 6)). Several attributes show unexpectedly low cell sizes for the -1 values (see Table 2), including attributes perceptions related to helping elders, correcting the young, being a member of a respected family, acting in ways that are good for the community, being a positive influence, helping people in need, and helping people who are left out. These low cell counts, representing areas around where it appears to be considerable disagreement, make the interpretation of perceptions of the attributes more difficult.

## Conclusion

Taken together, the positive associations between sociometric data on perceived attributes (or lack thereof) and data on perceived relationships (or lack thereof) lend support to the suggestion that rapid, bin-based sorting and clustering can lead to meaningful perceptual network data. Despite the limitations of the paper—small sample size, possible confusion on the part of respondents to the novel survey format, a lack of truly random recruitment—the methods described here provide a rapid, simple, and flexible means for producing sociometric representations based on cognitive network-style interviewing. By harnessing multiple reports on the presence or absence of ties between randomly chosen pairs, SNAPT data collection allows for “tomographic” approach to perceptual network data. As described above, analytical means are available that can rigorously evaluate this kind of data and render it in more classic sociometric form. Such results hold considerable promise for perceptual network analysis, given longstanding concerns over respondent reliability and network sampling.

Perhaps as importantly, the SNAPT method allows researchers to ascribe “robust intransitive” edges to some pairs—in the form of perceived negative ties. The presence of such negative ties in the network allows for more highly constrained group detection when compared with ordinary block modeling. As Mrvar [58] note: “[t]he implementation of constraints for partitioning signed networks is much more efficient than the one used for constraints in blockmodeling—it almost does not cost any additional time. Also, so called penalties are not needed anymore—partitions that do not fulfill constraints are simply ignored” [58]. More rigorous means for group detection have been a consistent goal of social network analysis [65].

In Table 3 we provide statistics of the social network induced by just the positive and negative edges. Although the summary statistics of these two networks are quite similar, we suspect that there are non-trivial relationships between the two overlapping edge sets. Indeed, the correlation coefficient of the positive and negative edge degrees of the set of vertices is 0.4875, confirming that the two types of edges are assigned to each vertex in a manner that is not uniformly random; nodes that have a higher number of positive edges tend somewhat to exhibit a higher number of negative edges as well. Exploring the structural relationships between these two co-occurring edge sets is a subject of ongoing research.

Lastly, it is worth noting again that the survey time and respondent burden associated with the “binning” of known (but not close to) alters was considerably less than ego-based data collection. Compared to a similar project carried out by the same research team in the Eastern Arctic [66, 67], interview times were reduced by more than 200%. As importantly, sample recruitment is not hampered by any required consideration of the underlying network topology. The value of such ease of implementation is contingent on the method producing meaningful data, of course. Our conclusion is that the present results provide some measure of support for such a claim.

Future work will involve the comparison of the perceived social network we obtained with the SNAPT method and the classical social networks. We also intend to investigate the effect of different choices with respect to the number of clusters *B*. In the present iteration of this research, we took *B* = 5 because we found that on average, between 15-20 of the 40 random pictures shown to each subject were classified as “recognized”, and thus, taking *B* = 5 bins allowed the mean occupancy of each bin to be between 3-4, close to the minimal size of a sociologically meaningful group. In future trials of this method, we will explore the effect of taking smaller or larger values of *B* (e.g. *B* = 3 or 7) on the number of clusters, as well as overall study conclusions. Furthermore, we will integrate the design of photo-capture and photo-weighting protocols for accumulating the “community of interest” from the sample itself. This capacity will allow for the use of SNAPT in unbounded communities.

## Supporting information

S1 Table Descriptive Statistics for the Northern Alaskan community network. (PDF)

S2 Table Multinomial Results: Makes positive changes in the community. (PDF)

S3 Table Multinomial Results: Helps young people in general. (PDF)

S4 Table Multinomial Results: Helps people with alcohol problems. (PDF)

S5 Table Multinomial Results: Helps women who are having trouble at home. (PDF)

S6 Table Multinomial Results: Helps men who are having trouble at home. (PDF)

S7 Table Multinomial Results: Helps elders who are having trouble at home. (PDF)

S8 Table Multinomial Results: Helps young people who are having trouble at home. (PDF)

S9 Table Multinomial Results: Helps people learn about traditional knowledge. (PDF)

S10 Table Multinomial Results: Gives money food or other needed things to people who need them. (PDF)

S11 Table Multinomial Results: Will correct a young person if he or she is doing something wrong. (PDF)

S12 Table Multinomial Results: Is a member of a respected family. (PDF)

S13 Table Multinomial Results: Act in ways that are good for the community. (PDF)

S14 Table Multinomial Results: Gives good advice most of the time. (PDF)

S15 Table Multinomial Results: Are a positive influence on others in this community. (PDF)

S16 Table Multinomial Results: Are willing to help out people who are in need. (PDF)

S17 Table Multinomial Results: Helps people who tend to be left out. (PDF)

S1 File The individual survey data, edge properties, and codebook are provided in S1_File.zip. (ZIP)

## References

[1] Freeman LC. Filling in the blanks: A theory of cognitive categories and the structure of social affiliation. Social Psychology Quarterly. 1992; p. 118–127. 10.2307/2786941

[2] Brashears ME, Brashears LA. The Enemy of My Friend Is Easy to Remember: Balance as a Compression Heuristic In: Advances in Group Processes. Emerald Group Publishing Limited; 2016 p. 1–31.

[3] Krackhardt D. Cognitive social structures. Social networks. 1987;9(2):109–134. 10.1016/0378-8733(87)90009-8

[4] Neal JW. “Kracking” the missing data problem: applying Krackhardt’s cognitive social structures to school-based social networks. Sociology of Education. 2008;81(2):140–162. 10.1177/003804070808100202

[5] Brashears ME, Quintane E. The microstructures of network recall: How social networks are encoded and represented in human memory. Social Networks. 2015;41:113–126. 10.1016/j.socnet.2014.11.003

[6] Brands RA. Cognitive social structures in social network research: A review. Journal of Organizational Behavior. 2013;34(S1). 10.1002/job.1890

[7] Luria G, Kalish Y. A social network approach to peer assessment: Improving predictive validity. Human Resource Management. 2013;52(4):537–560. 10.1002/hrm.21541

[8] Marsden PV. Interviewer effects in measuring network size using a single name generator. Social Networks. 2003;25(1):1–16. 10.1016/S0378-8733(02)00009-6

[9] Heckathorn DD, Cameron CJ. Network Sampling. Annual Review of Sociology. 2017;43(1). 10.1146/annurev-soc-060116-053556

[10] Erickson BH, Nosanchuk TA. Applied network sampling. Social Networks. 1983;5(4):367–382. 10.1016/0378-8733(83)90008-4

[11] Bernard HR, Killworth P, Kronenfeld D, Sailer L. The problem of informant accuracy: The validity of retrospective data. Annual review of anthropology. 1984;13(1):495–517. 10.1146/annurev.an.13.100184.002431

[12] Robins G. Doing social network research: Network-based research design for social scientists. Sage; 2015.

[13] Barnes JA. Class and committees in a Norwegian island parish. Human relations. 1954;7(1):39–58. 10.1177/001872675400700102

[14] Mitchell JC. Social networks. Annual review of anthropology. 1974;3(1):279–299. 10.1146/annurev.an.03.100174.001431

[15] Prell C. Social network analysis: History, theory and methodology. Sage; 2012.

[16] Otte E, Rousseau R. Social network analysis: a powerful strategy, also for the information sciences. Journal of information Science. 2002;28(6):441–453. 10.1177/016555150202800601

[17] Lewis K, Kaufman J, Gonzalez M, Wimmer A, Christakis N. Tastes, ties, and time: A new social network dataset using Facebook. com. Social networks. 2008;30(4):330–342. 10.1016/j.socnet.2008.07.002

[18] Smith JA. Macrostructure from microstructure: Generating whole systems from ego networks. Sociological methodology. 2012;42(1):155–205. 10.1177/0081175012455628 25339783 PMC4203462

[19] Frank O. Sampling and estimation in large social networks. Social networks. 1978;1(1):91–101. 10.1016/0378-8733(78)90015-1

[20] Frank O. Survey sampling in graphs. Journal of Statistical Planning and Inference. 1977;1(3):235–264. 10.1016/0378-3758(77)90011-8

[21] Frank O. Network sampling and model fitting. Models and methods in social network analysis. 2005; p. 31–56. 10.1017/CBO9780511811395.003

[22] Ma H, Gustafson S, Moitra A, Bracewell D. Ego-centric network sampling in viral marketing applications. In: Computational Science and Engineering, 2009. CSE’09. International Conference on. vol. 4. IEEE; 2009. p. 777–782.

[23] Campbell KE, Lee BA. Name generators in surveys of personal networks. Social networks. 1991;13(3):203–221. 10.1016/0378-8733(91)90006-F

[24] Marsden PV. Core discussion networks of Americans. American sociological review. 1987; p. 122–131. 10.2307/2095397

[25] Bearman PS, Moody J, Stovel K. Chains of affection: The structure of adolescent romantic and sexual networks 1. American journal of sociology. 2004;110(1):44–91. 10.1086/386272

[26] Goodreau SM. Advances in exponential random graph (p*) models applied to a large social network. Social Networks. 2007;29(2):231–248. 10.1016/j.socnet.2006.08.001 18449326 PMC2031833

[27] Carpenter MA, Li M, Jiang H. Social network research in organizational contexts: A systematic review of methodological issues and choices. Journal of Management. 2012;38(4):1328–1361. 10.1177/0149206312440119

[28] Provan KG, Fish A, Sydow J. Interorganizational networks at the network level: A review of the empirical literature on whole networks. Journal of management. 2007;33(3):479–516. 10.1177/0149206307302554

[29] Dombrowski K, Khan B, Moses J, Channell E, Misshula E, et al Assessing respondent driven sampling for network studies in ethnographic contexts. Advances in Anthropology. 2013;3(01):1 10.4236/aa.2013.31001

[30] Wejnert C. Social network analysis with respondent-driven sampling data: A study of racial integration on campus. Social Networks. 2010;32(2):112–124. 10.1016/j.socnet.2009.09.002 20383316 PMC2850221

[31] Browne K. Snowball sampling: using social networks to research non-heterosexual women. International journal of social research methodology. 2005;8(1):47–60. 10.1080/1364557032000081663

[32] Voorhees CC, Murray D, Welk G, Birnbaum A, Ribisl KM, Johnson CC, et al The role of peer social network factors and physical activity in adolescent girls. American journal of health behavior. 2005;29(2):183–190. 10.5993/AJHB.29.2.9 15698985 PMC2507875

[33] Matzat U, Snijders C. Does the online collection of ego-centered network data reduce data quality? An experimental comparison. Social Networks. 2010;32(2):105–111. 10.1016/j.socnet.2009.08.002

[34] Khan B, Dombrowski K, Curtis R, Wendel T. Estimating Vertex Measures in Social Networks by Sampling Completions of RDS Trees. Social networking. 2015;4(1):1 10.4236/sn.2015.41001 25838988 PMC4380167

[35] Smith JA, Moody J. Structural effects of network sampling coverage I: Nodes missing at random. Social networks. 2013;35(4):652–668. 10.1016/j.socnet.2013.09.003 PMC3846431 24311893

[36] Kossinets G. Effects of missing data in social networks. Social networks. 2006;28(3):247–268. 10.1016/j.socnet.2005.07.002

[37] Fründ J, McCann KS, Williams NM. Sampling bias is a challenge for quantifying specialization and network structure: lessons from a quantitative niche model. Oikos. 2016;125(4):502–513. 10.1111/oik.02256

[38] McCarty C, Killworth PD, Rennell J. Impact of methods for reducing respondent burden on personal network structural measures. Social networks. 2007;29(2):300–315. 10.1016/j.socnet.2006.12.005

[39] Brewer DD. Forgetting in the recall-based elicitation of personal and social networks. Social networks. 2000;22(1):29–43. 10.1016/S0378-8733(99)00017-9

[40] Bernard HR, Johnsen EC, Killworth PD, McCarty C, Shelley GA, Robinson S. Comparing four different methods for measuring personal social networks. Social networks. 1990;12(3):179–215. 10.1016/0378-8733(90)90005-T

[41] Knoke D, Yang S. Social network analysis. vol. 154 Sage; 2008.

[42] Wasserman S, Faust K. Social network analysis: Methods and applications. vol. 8 Cambridge university press; 1994.

[43] Newman ME, Girvan M. Finding and evaluating community structure in networks. Physical review E. 2004;69(2):026113 10.1103/PhysRevE.69.026113 14995526

[44] Newman ME. Modularity and community structure in networks. Proceedings of the national academy of sciences. 2006;103(23):8577–8582. 10.1073/pnas.0601602103 PMC1482622 16723398

[45] Fortunato S. Community detection in graphs. Physics reports. 2010;486(3):75–174. 10.1016/j.physrep.2009.11.002

[46] Reichardt J, Bornholdt S. Statistical mechanics of community detection. Physical Review E. 2006;74(1):016110 10.1103/PhysRevE.74.016110 16907154

[47] Traag VA, Bruggeman J. Community detection in networks with positive and negative links. Physical Review E. 2009;80(3):036115 10.1103/PhysRevE.80.036115 19905188

[48] Mucha PJ, Richardson T, Macon K, Porter MA, Onnela JP. Community structure in time-dependent, multiscale, and multiplex networks. science. 2010;328(5980):876–878. 10.1126/science.1184819 20466926

[49] Heider F. Attitudes and cognitive organization. The Journal of psychology. 1946;21(1):107–112. 10.1080/00223980.1946.9917275 21010780

[50] Cartwright D, Harary F. Structural balance: a generalization of Heider’s theory. Psychological review. 1956;63(5):277 10.1037/h0046049 13359597

[51] Davis JA. Clustering and structural balance in graphs. Human relations. 1967;20(2):181–187. 10.1177/001872676702000206

[52] Doreian P, Mrvar A. Testing two theories for generating signed networks using real data. Metodoloski Zvezki. 2014;11(1):31.

[53] Doreian P, Kapuscinski R, Krackhardt D, Szczypula J. A brief history of balance through time. Journal of Mathematical Sociology. 1996;21(1-2):113–131. 10.1080/0022250X.1996.9990176

[54] Leskovec J, Huttenlocher D, Kleinberg J. Predicting positive and negative links in online social networks. In: Proceedings of the 19th international conference on World wide web. ACM; 2010. p. 641–650.

[55] Newcomb T. The Acquaintance Process. New York: Holt, Rinehart and Winston. (1966).“The General Nature of Peer Group Influence”, pps. 2-16 in College Peer Groups, edited by TM Newcomb and EK Wilson; 1961.

[56] Wexler L, McEachern D, DiFulvio G, Smith C, Graham LF, Dombrowski K. Creating a community of practice to prevent suicide through multiple channels: describing the theoretical foundations and structured learning of PC CARES. International quarterly of community health education. 2016;36(2):115–122. 10.1177/0272684X16630886 26880738 PMC4794395

[57] Dombrowski K, Channell E, Khan B, Moses J, Misshula E. Out on the land: Income, subsistence activities, and food sharing networks in Nain, Labrador. Journal of Anthropology. 2013;2013 10.1155/2013/185048

[58] Mrvar A, Doreian P. Partitioning signed networks using constraints.; 2015. Available from: http://mrvar.fdv.uni-lj.si/pajek/SignedNetworks/UsingConstraints.pdf.

[59] De Nooy W, Mrvar A, Batagelj V. Exploratory social network analysis with Pajek.; 2011.

[60] Batagelj V, Mrvar A. Pajek manual 4.10.; 2016.

[61] Simmel G. The number of members as determining the sociological form of the group. I. American Journal of Sociology. 1902;8(1):1–46. 10.1086/211115

[62] van Rijsewijk L, Dijkstra JK, Pattiselanno K, Steglich C, Veenstra R. Who helps whom? Investigating the development of adolescent prosocial relationships. Developmental psychology. 2016;52(6):894 10.1037/dev0000106 27228450

[63] Centola D, Gonzalez-Avella JC, Eguiluz VM, San Miguel M. Homophily, cultural drift, and the co-evolution of cultural groups. Journal of Conflict Resolution. 2007;51(6):905–929. 10.1177/0022002707307632

[64] Long JS, Freese J. Regression models for categorical dependent variables using Stata. Stata press; 2006.

[65] Freeman L. The development of social network analysis. A Study in the Sociology of Science. 2004;1.

[66] Dombrowski K, Khan B, Moses J, Channell E, Dombrowski N. Network sampling of social divisions in a rural inuit community. Identities. 2014;21(2):134–151. 10.1080/1070289X.2013.854718

[67] Dombrowski K, Habecker P, Gauthier GR, Khan B, Moses J, Arzyutov DV, et al Relocation Redux: Labrador Inuit Population Movements and Inequalities in the Land Claims Era. Current Anthropology. 2016;57(6):000–000. 10.1086/689210

