# Supplementary material for: Mapping the structure of perceptions in helping networks of Alaska Natives
Source: PLoS One. 2018 Nov 12;13(11):e0204343. doi: 10.1371/journal.pone.0204343 (PMC6231607; doi:10.1371/journal.pone.0204343)
Supplement: S1 Table — (PDF) [file pone.0204343.s001.pdf]

S1 Table. Descriptive Statistics For the Northern Alaskan community network

Variable	N	Mean/%	Std. Dev.	Min	Max
Female	167	41%	0.49	0	1
Age	169	35.42	17.45	11	89
Education					
8th or Less	162	10%	0.31	0	1
Some HS	162	20%	0.4	0	1
HS Complete	162	57%	0.5	0	1
Some College	162	9%	0.29	0	1
2 Year Degree	162	2%	0.14	0	1
4 Year Degree	162	1%	0.08	0	1
Graduate Work	162	1%	0.08	0	1
Weekly Household Income					
$100	143	31%	0.47	0	1
$101-200	143	13%	0.34	0	1
$201-400	143	15%	0.36	0	1
$401-600	143	6%	0.24	0	1
$601-1000	143	13%	0.34	0	1
$1000+	143	20%	0.4	0	1
Weekly Individual Income					
$100	127	42%	0.5	0	1
$101-200	127	11%	0.31	0	1
$201-400	127	13%	0.33	0	1
$401-600	127	9%	0.28	0	1
$601-1000	127	13%	0.34	0	1
$1000+	127	13%	0.33	0	1
Number of Children in Household	166	2.42	2.03	0	9
Number of Adults in household	168	2.92	1.5	0	7
Ratio of People to Bedrooms	160	1.99	1.33	0	12
Subsistence Access Scale[a]	162	2.51	2.04	0	6

[a] - *Local Measure for Socioeconomic Status*
