# Supplementary material for: Mapping the structure of perceptions in helping networks of Alaska Natives
Source: PLoS One. 2018 Nov 12;13(11):e0204343. doi: 10.1371/journal.pone.0204343 (PMC6231607; doi:10.1371/journal.pone.0204343)
Supplement: S2 Table — (PDF) [file pone.0204343.s002.pdf]

S2 Table. Multinomial Results: Makes positive changes in the community

	Dependent variable:	
	Makes positive changes in the community[a]	
	(-1)	(1)
Class 1[b]	−8.542	−0.106
	(98.995)	(0.588)
Class 2[b]	−7.007	0.871*
	(47.379)	(0.445)
Class 4[b]	0.048	−0.147
	(1.234)	(0.475)
Class 5[b]	0.511	−0.243
	(1.238)	(0.585)
Class 6[b]	1.313	−0.135
	(1.018)	(0.587)
Constant	−4.175***	−2.034***
	(0.713)	(0.258)
Akaike Inf. Crit.	355.913	355.913

**p<0.1; **p<0.05; ***p<0.01*

[a] *- Reference category - "0"s*

[b] *- Reference category - Class 3*
