# Supplementary material for: Mapping the structure of perceptions in helping networks of Alaska Natives
Source: PLoS One. 2018 Nov 12;13(11):e0204343. doi: 10.1371/journal.pone.0204343 (PMC6231607; doi:10.1371/journal.pone.0204343)
Supplement: S3 Table — (PDF) [file pone.0204343.s003.pdf]

S3 Table. Multinomial Results: Helps young people in general

	Dependent variable:	
	Helps young people in general[a]	
	(-1)	(1)
Class 1[b]	−6.036	−0.198
	(40.054)	(0.664)
Class 2[b]	1.316	0.285
	(1.427)	(0.554)
Class 4[b]	0.741	−0.290
	(1.423)	(0.543)
Class 5[b]	1.289	0.440
	(1.427)	(0.522)
Class 6[b]	1.344	0.313
	(1.428)	(0.555)
Constant	−4.900***	−2.259***
	(1.005)	(0.281)
Akaike Inf. Crit.	319.711	319.711

*p<0.1; **p<0.05; ***p<0.01

[a] *- Reference category - "0"s*

[b] *- Reference category - Class 3*
