# Supplementary material for: Mapping the structure of perceptions in helping networks of Alaska Natives
Source: PLoS One. 2018 Nov 12;13(11):e0204343. doi: 10.1371/journal.pone.0204343 (PMC6231607; doi:10.1371/journal.pone.0204343)
Supplement: S4 Table — (PDF) [file pone.0204343.s004.pdf]

S4 Table. Multinomial Results: Helps people with alcohol problems

	Dependent variable:	
	Helps people with alcohol problems[a]	
	(-1)	(1)
Class 1[b]	0.700	0.006
	(0.886)	(1.134)
Class 2[b]	1.050	0.644
	(0.786)	(0.885)
Class 4[b]	0.065	0.065
	(0.879)	(0.879)
Class 5[b]	0.518	−10.160
	(0.883)	(147.243)
Class 6[b]	0.644	0.644
	(0.885)	(0.885)
Constant	-3.562^{***}	-3.562^{***}
	(0.507)	(0.507)
Akaike Inf. Crit.	243.888	243.888

$^{*}p<0.1$; $^{**}p<0.05$; $^{***}p<0.01$

[a] *- Reference category - "0"s*

[b] *- Reference category - Class 3*
