# Supplementary material for: Mapping the structure of perceptions in helping networks of Alaska Natives
Source: PLoS One. 2018 Nov 12;13(11):e0204343. doi: 10.1371/journal.pone.0204343 (PMC6231607; doi:10.1371/journal.pone.0204343)
Supplement: S5 Table — (PDF) [file pone.0204343.s005.pdf]

S5 Table. Multinomial Results: Helps women who are having trouble at home

	Dependent variable:	
	Helps women who are having trouble at home[a]	
	(-1)	(1)
Class 1[b]	−0.609	−0.273
	(1.085)	(1.111)
Class 2[b]	0.777	0.420
	(0.654)	(0.857)
Class 4[b]	−0.552	−15.156
	(0.815)	(1, 240.691)
Class 5[b]	0.359	−33.799***
	(0.713)	(0.000)
Class 6[b]	−0.663	0.366
	(1.085)	(0.857)
Constant	−2.974***	−3.311***
	(0.388)	(0.455)
Akaike Inf. Crit.	249.647	249.647

**p<0.1; **p<0.05; ***p<0.01*

[a] *- Reference category - "0"s*

[b] *- Reference category - Class 3*
