# Supplementary material for: Mapping the structure of perceptions in helping networks of Alaska Natives
Source: PLoS One. 2018 Nov 12;13(11):e0204343. doi: 10.1371/journal.pone.0204343 (PMC6231607; doi:10.1371/journal.pone.0204343)
Supplement: S6 Table — (PDF) [file pone.0204343.s006.pdf]

S6 Table. Multinomial Results: Helps men who are having trouble at home

	Dependent variable:	
	Helps men who are having trouble at home[a]	
	(-1)	(1)
Class 1[b]	-19.740^{***}	-17.726^{***}
	(0.00000)	(0.00001)
Class 2[b]	0.064	1.029
	(0.823)	(0.786)
Class 4[b]	-0.125	-14.984
	(0.706)	(1,287.220)
Class 5[b]	0.391	-0.148
	(0.713)	(1.132)
Class 6[b]	-17.594^{***}	0.571
	(0.00000)	(0.885)
Constant	-2.981^{***}	-3.541^{***}
	(0.387)	(0.507)
Akaike Inf. Crit.	226.371	226.371

**p*<0.1; ***p*<0.05; ****p*<0.01

[a] *- Reference category - "0"s*

[b] *- Reference category - Class 3*
