# Supplementary material for: Mapping the structure of perceptions in helping networks of Alaska Natives
Source: PLoS One. 2018 Nov 12;13(11):e0204343. doi: 10.1371/journal.pone.0204343 (PMC6231607; doi:10.1371/journal.pone.0204343)
Supplement: S7 Table — (PDF) [file pone.0204343.s007.pdf]

S7 Table. Multinomial Results: Helps elders who are having trouble at home

	Dependent variable:	
	Helps elders who are having trouble at home[a]	
	(-1)	(1)
Class 1[b]	−10.413	0.436
	(370.641)	(0.518)
Class 2[b]	1.299	0.136
	(1.427)	(0.546)
Class 4[b]	−82.319	−0.257
		(0.502)
Class 5[b]	−12.151	−0.505
	(780.589)	(0.654)
Class 6[b]	−11.732	0.530
	(695.333)	(0.492)
Constant	−4.883***	−2.110***
	(1.004)	(0.265)
Akaike Inf. Crit.	310.731	310.731

**p<0.1; **p<0.05; ***p<0.01*

[a] *- Reference category - "0"s*

[b] *- Reference category - Class 3*
