# Supplementary material for: Mapping the structure of perceptions in helping networks of Alaska Natives
Source: PLoS One. 2018 Nov 12;13(11):e0204343. doi: 10.1371/journal.pone.0204343 (PMC6231607; doi:10.1371/journal.pone.0204343)
Supplement: S8 Table — (PDF) [file pone.0204343.s008.pdf]

S8 Table. Multinomial Results: Helps young people who are having trouble at home

	Dependent variable:	
	Helps young people who are having trouble at home[a]	
	(-1)	(1)
Class 1[b]	−10.069	0.583
	(178.046)	(0.631)
Class 2[b]	0.265	0.959*
	(1.170)	(0.560)
Class 4[b]	−0.368	−0.368
	(1.164)	(0.683)
Class 5[b]	0.107	−0.298
	(1.168)	(0.802)
Class 6[b]	−9.075	0.498
	(103.822)	(0.629)
Constant	−3.821***	−2.723***
	(0.584)	(0.344)
Akaike Inf. Crit.	277.223	277.223

*p<0.1; **p<0.05; ***p<0.01

[a] - Reference category - ”0”s

[b] - Reference category - Class 3
