# Supplementary material for: Mapping the structure of perceptions in helping networks of Alaska Natives
Source: PLoS One. 2018 Nov 12;13(11):e0204343. doi: 10.1371/journal.pone.0204343 (PMC6231607; doi:10.1371/journal.pone.0204343)
Supplement: S9 Table — (PDF) [file pone.0204343.s009.pdf]

S9 Table. Multinomial Results: Helps people learn about traditional knowledge

	Dependent variable:	
	Helps people learn about traditional knowledge[a]	
	(-1)	(1)
Class 1[b]	−11.592	−11.203
	(428.801)	(117.623)
Class 2[b]	0.767	0.967**
	(1.241)	(0.433)
Class 4[b]	0.089	0.195
	(1.235)	(0.425)
Class 5[b]	−8.934	−0.645
	(109.284)	(0.649)
Class 6[b]	0.531	−0.975
	(1.238)	(0.768)
Constant	−4.168***	−1.969***
	(0.713)	(0.252)
Akaike Inf. Crit.	326.434	326.434

*p<0.1; **p<0.05; ***p<0.01

[a] - Reference category - "0"s

[b] - Reference category - Class 3
