# Supplementary material for: Mapping the structure of perceptions in helping networks of Alaska Natives
Source: PLoS One. 2018 Nov 12;13(11):e0204343. doi: 10.1371/journal.pone.0204343 (PMC6231607; doi:10.1371/journal.pone.0204343)
Supplement: S10 Table — (PDF) [file pone.0204343.s010.pdf]

S10 Table. Multinomial Results: Gives money food or other needed things to people who need them

	Dependent variable:	
	Gives money food or other needed things to people who need them[a]	
	(-1)	(1)
Class 1[b]	-7.001	-0.037
	(46.001)	(0.591)
Class 2[b]	1.509	1.133^{**}
	(1.021)	(0.442)
Class 4[b]	0.748	-0.232
	(1.012)	(0.503)
Class 5[b]	0.599	0.465
	(1.239)	(0.491)
Class 6[b]	0.781	1.004^{**}
	(1.241)	(0.451)
Constant	-4.182^{***}	-2.103^{***}
	(0.712)	(0.265)
Akaike Inf. Crit.	395.981	395.981

*p<0.1; **p<0.05; ***p<0.01

[a] - Reference category - ”0”s

[b] - Reference category - Class 3
