# Supplementary material for: Mapping the structure of perceptions in helping networks of Alaska Natives
Source: PLoS One. 2018 Nov 12;13(11):e0204343. doi: 10.1371/journal.pone.0204343 (PMC6231607; doi:10.1371/journal.pone.0204343)
Supplement: S11 Table — (PDF) [file pone.0204343.s011.pdf]

S11 Table. Multinomial Results: Will correct a young person if he or she is doing something wrong

	Dependent variable:	
	Will correct a young person if he or she is doing something wrong[a]	
	(-1)	(1)
Class 1[b]	−6.542	0.119
	(52.738)	(0.431)
Class 2[b]	−5.789	0.595
	(36.827)	(0.383)
Class 4[b]	0.731	−0.173
	(1.424)	(0.367)
Class 5[b]	−6.193	−0.071
	(40.313)	(0.424)
Class 6[b]	1.321	0.051
	(1.429)	(0.428)
Constant	−4.755***	−1.288***
	(1.005)	(0.200)
Akaike Inf. Crit.	459.511	459.511

$^{*}p<0.1$; $^{**}p<0.05$; $^{***}p<0.01$

[a] *- Reference category - "0"s*

[b] *- Reference category - Class 3*
