# Supplementary material for: Mapping the structure of perceptions in helping networks of Alaska Natives
Source: PLoS One. 2018 Nov 12;13(11):e0204343. doi: 10.1371/journal.pone.0204343 (PMC6231607; doi:10.1371/journal.pone.0204343)
Supplement: S12 Table — (PDF) [file pone.0204343.s012.pdf]

S12 Table. Multinomial Results: Is a member of a respected family

	Dependent variable:	
	Is a member of a respected family[a]	
	(-1)	(1)
Class 1[b]	-4.574^{***}	0.152
	(0.000)	(0.385)
Class 2[b]	-5.055^{***}	0.321
	(0.000)	(0.364)
Class 4[b]	-8.425^{***}	-0.331
	(0.000)	(0.330)
Class 5[b]	-5.296^{***}	-0.175
	(0.000)	(0.382)
Class 6[b]	18.958^{***}	-0.163
	(0.509)	(0.396)
Constant	-22.325^{***}	-0.806^{***}
	(0.509)	(0.177)
Akaike Inf. Crit.	498.460	498.460

$^{*}p<0.1$; $^{**}p<0.05$; $^{***}p<0.01$

[a] - Reference category - "0"s

[b] - Reference category - Class 3
