# Supplementary material for: Mapping the structure of perceptions in helping networks of Alaska Natives
Source: PLoS One. 2018 Nov 12;13(11):e0204343. doi: 10.1371/journal.pone.0204343 (PMC6231607; doi:10.1371/journal.pone.0204343)
Supplement: S13 Table — (PDF) [file pone.0204343.s013.pdf]

S13 Table. Multinomial Results: Act in ways that are good for the community

	Dependent variable:	
	Act in ways that are good for the community[a]	
	(-1)	(1)
Class 1[b]	5.883***	0.037
	(0.00003)	(0.401)
Class 2[b]	23.893***	0.489
	(5.348)	(0.368)
Class 4[b]	17.075	−0.053
	(15.871)	(0.325)
Class 5[b]	8.746***	0.066
	(0.001)	(0.377)
Class 6[b]	24.339***	−0.585
	(5.324)	(0.455)
Constant	−27.111***	−0.935***
	(5.299)	(0.182)
Akaike Inf. Crit.	499.214	499.214

**p<0.1; **p<0.05; ***p<0.01*

[a] - Reference category - "0"s

[b] - Reference category - Class 3
