# Supplementary material for: Mapping the structure of perceptions in helping networks of Alaska Natives
Source: PLoS One. 2018 Nov 12;13(11):e0204343. doi: 10.1371/journal.pone.0204343 (PMC6231607; doi:10.1371/journal.pone.0204343)
Supplement: S14 Table — (PDF) [file pone.0204343.s014.pdf]

S14 Table. Multinomial Results: Gives good advice most of the time

	Dependent variable:	
	Gives good advice most of the time[a]	
	(-1)	(1)
Class 1[b]	−9.761	0.071
	(261.318)	(0.501)
Class 2[b]	2.064*	0.359
	(1.241)	(0.458)
Class 4[b]	−56.242	−0.622
		(0.485)
Class 5[b]	1.892	−0.506
	(1.239)	(0.574)
Class 6[b]	−10.618	−0.018
	(383.588)	(0.499)
Constant	−4.836***	−1.745***
	(1.004)	(0.231)
Akaike Inf. Crit.	367.467	367.467

**p<0.1; **p<0.05; ***p<0.01*

[a] *- Reference category - "0"s*

[b] *- Reference category - Class 3*
