# Supplementary material for: Mapping the structure of perceptions in helping networks of Alaska Natives
Source: PLoS One. 2018 Nov 12;13(11):e0204343. doi: 10.1371/journal.pone.0204343 (PMC6231607; doi:10.1371/journal.pone.0204343)
Supplement: S15 Table — (PDF) [file pone.0204343.s015.pdf]

S15 Table. Multinomial Results: Are a positive influence on others in this community

	Dependent variable:	
	Are a positive influence on others in this community[a]	
	(-1)	(1)
Class 1[b]	−11.639	−0.227
	(459.336)	(0.492)
Class 2[b]	−10.106	0.284
	(213.373)	(0.419)
Class 4[b]	−13.647	−0.467
	(907.763)	(0.414)
Class 5[b]	0.531	−0.029
	(1.240)	(0.445)
Class 6[b]	0.589	−0.104
	(1.240)	(0.466)
Constant	−4.086***	−1.447***
	(0.713)	(0.210)
Akaike Inf. Crit.	418.699	418.699

*p<0.1; **p<0.05; ***p<0.01

[a] - Reference category - "0"s

[b] - Reference category - Class 3
