# Supplementary material for: Mapping the structure of perceptions in helping networks of Alaska Natives
Source: PLoS One. 2018 Nov 12;13(11):e0204343. doi: 10.1371/journal.pone.0204343 (PMC6231607; doi:10.1371/journal.pone.0204343)
Supplement: S16 Table — (PDF) [file pone.0204343.s016.pdf]

S16 Table. Multinomial Results: Are willing to help out people who are in need

	Dependent variable:	
	Are willing to help out people who are in need[a]	
	(-1)	(1)
Class 1[b]	1.859	−0.333
	(2.349)	(0.421)
Class 2[b]	5.609	0.452
	(329.110)	(0.361)
Class 4[b]	9.458	−0.379
	(118.575)	(0.336)
Class 5[b]	2.941	−0.520
	(8.829)	(0.414)
Class 6[b]	17.527	−0.713
	(114.309)	(0.453)
Constant	−21.024	−0.838***
	(114.307)	(0.178)
Akaike Inf. Crit.	471.180	471.180

$^{*}p<0.1$; $^{**}p<0.05$; $^{***}p<0.01$

[a] *- Reference category - "0"s*

[b] *- Reference category - Class 3*
