# Supplementary material for: Mapping the structure of perceptions in helping networks of Alaska Natives
Source: PLoS One. 2018 Nov 12;13(11):e0204343. doi: 10.1371/journal.pone.0204343 (PMC6231607; doi:10.1371/journal.pone.0204343)
Supplement: S17 Table — (PDF) [file pone.0204343.s017.pdf]

S17 Table. Multinomial Results: Helps people who tend to be left out

	Dependent variable:
	Helps people who tend to be left out[a]
Class 1[b]	0.507
	(0.366)
Class 2[b]	0.412
	(0.352)
Class 4[b]	−0.081
	(0.301)
Class 5[b]	−0.152
	(0.360)
Class 6[b]	−0.496
	(0.391)
Constant	−0.507***
	(0.169)
Akaike Inf. Crit.	515.479

**p<0.1; **p<0.05; ***p<0.01*

[a] - Reference category - "0"s

[b] - Reference category - Class 3
